# Elevated Intraocular Pressure Causes Abnormal Reactivity of Mouse Retinal Arterioles

**DOI:** 10.1155/2019/9736047

**Published:** 2019-12-29

**Authors:** Adrian Gericke, Carolina Mann, Jenia Kouchek Zadeh, Aytan Musayeva, Ismael Wolff, Maoren Wang, Norbert Pfeiffer, Andreas Daiber, Huige Li, Ning Xia, Verena Prokosch

**Affiliations:** ^1^Department of Ophthalmology, University Medical Center, Johannes Gutenberg University Mainz, Langenbeckstr. 1, 55131 Mainz, Germany; ^2^Center for Cardiology 1, Molecular Cardiology, University Medical Center, Johannes Gutenberg University Mainz, Langenbeckstr. 1, 55131 Mainz, Germany; ^3^Department of Pharmacology, University Medical Center, Johannes Gutenberg University Mainz, Obere Zahlbacher Str. 67, 55131 Mainz, Germany

## Abstract

**Objective:**

Glaucoma is a leading cause of severe visual impairment and blindness. Although high intraocular pressure (IOP) is an established risk factor for the disease, the role of abnormal ocular vessel function in the pathophysiology of glaucoma gains more and more attention. We tested the hypothesis that elevated intraocular pressure (IOP) causes vascular dysfunction in the retina.

**Methods:**

High IOP was induced in one group of mice by unilateral cauterization of three episcleral veins. The other group received sham surgery only. Two weeks later, retinal vascular preparations were studied by video microscopy in vitro. Reactive oxygen species (ROS) levels and expression of hypoxia markers and of prooxidant and antioxidant redox genes as well as of inflammatory cytokines were determined.

**Results:**

Strikingly, responses of retinal arterioles to stepwise elevation of perfusion pressure were impaired in the high-IOP group. Moreover, vasodilation responses to the endothelium-dependent vasodilator, acetylcholine, were markedly reduced in mice with elevated IOP, while no differences were seen in response to the endothelium-independent nitric oxide donor, sodium nitroprusside. Remarkably, ROS levels were increased in the retinal ganglion cell layer including blood vessels. Expression of the NADPH oxidase isoform, NOX2, and of the inflammatory cytokine, TNF-*α*, was increased at the mRNA level in retinal explants. Expression of NOX2, but not of the hypoxic markers, HIF-1*α* and VEGF-A, was increased in the retinal ganglion cell layer and in retinal blood vessels at the protein level.

**Conclusion:**

Our data provide first-time evidence that IOP elevation impairs autoregulation and induces endothelial dysfunction in mouse retinal arterioles. Oxidative stress and inflammation, but not hypoxia, appear to be involved in this process.

## 1. Introduction

Glaucoma is a leading cause of blindness characterized by progressive loss of retinal ganglion cells (RGC) and their axons [1]. Elevated intraocular pressure (IOP) is a major risk factor for the onset and progression of the disease, although other risk factors, such as vascular dysfunction/dysregulation and autoimmunological mechanisms, have also been implicated in the pathophysiology [2–4]. Whether the individual risk factors are independent of each other is unknown at present. Intriguingly, it has been shown in patients that glaucoma may progress despite normalization of IOP [5]. Thus, once initiated, molecular and morphological events, which are poorly understood, appear to take place independently from acute IOP levels. It has been demonstrated that elevated IOP causes oxidative stress, inflammation, and consequently tissue damage in the retina and optic nerve [6]. Support for a link between oxidative stress and ocular vascular damage in glaucoma comes from studies reporting that levels of reactive oxygen species (ROS) and of markers for vascular endothelial function are increased in glaucoma patients both in aqueous humor and blood serum [7–10]. Moreover, optic disc hemorrhage and reduced peripapillary and macular microvascular density are typical findings in glaucoma patients suggesting that vascular integrity is compromised in the disease [11–15]. However, it is debated whether elevated IOP stresses neurons and glial cells only and abnormal vascular function is an additional IOP-independent factor for glaucoma or whether abnormally high IOP levels induce alterations in vascular cells, which may promote further neuron damage by disturbances of ocular perfusion [16–19]. In the present study, we experimentally increased IOP in mice to examine vascular function in the retina. We tested, for the first time, whether there is a direct interaction between IOP and vascular responsiveness in the retina. Our hypothesis was that elevated IOP induces abnormal reactivity in retinal arterioles. Another objective of this study was to examine the role of oxidative stress in this context.

## 2. Materials and Methods

### 2.1. Animals

All experiments were approved by the Animal Care Committee of Rhineland-Palatinate, Germany, and all animals were treated in accordance with the EU Directive 2010/63/EU for animal experiments. Male mice (C57BL/6J) at the age of 8-10 weeks were used for experiments. Mice were housed under standardized conditions with a 12 h light/dark cycle, temperature of 22 ± 2°C, humidity of 55 ± 10%, and free access to food and tap water.

### 2.2. Induction of High Intraocular Pressure

Increased IOP was induced in one eye per mouse by cauterization of three episcleral veins as shown previously [20]. Under anesthesia with ketamine/xylazine, the conjunctiva was carefully incised, and the veins were exposed and cauterized using a battery cauter (Faromed GmbH Medizintechnik, Berlin, Germany). The conjunctiva was then pulled back in place, and antibiotic ointment (ofloxacin 3 mg/g, Bausch & Lomb GmbH, Berlin, Germany) was topically administered. The sham group underwent a very similar surgical procedure as the cauterized group; however, after incision of the conjunctiva, the episcleral veins were exposed and just touched with the tip of the cauter without applying heat. The conjunctiva was pulled back and antibiotic ointment topically applied, such as in the cauterized eyes.

### 2.3. Measurement of Intraocular Pressure and Blood Pressure

The Icare® TONOLAB rebound tonometer (Bon Optic, Lübeck, Germany) was used for the measurement of IOP in conscious restrained mice. Topical anesthesia with proparacaine 0.5% (URSAPHARM Arzneimittel GmbH, Saarbrücken, Germany) was used directly before each examination. Ten IOP measurements were taken each time per eye from which the mean was calculated. Blood pressure measurements with a computerized tail-cuff system (CODA® Monitor, Kent Scientific) were conducted in conscious restrained mice. Before measurement, mice were trained for two consecutive days to become used to the procedure. Mice were placed in restraint tubes to prevent excessive movement during measurement and placed on a warming platform (32-35°C). After tails had been cuffed, mice were allowed to settle for 5 minutes before the start of the experiment. Each session consisted of 20 measuring cycles, of which the first 5 cycles were used for acclimatization and were excluded from analysis. The average of the following 15 cycles was taken as the reading for each mouse. All measurements of IOP and blood pressure were conducted in the morning between 9:00 a.m. and 12:00 a.m. right before surgery and 7 and 14 days after surgery. Ocular perfusion pressure (OPP) was calculated as the difference between mean arterial pressure (MAP) and IOP.

### 2.4. Measurement of Vascular Reactivity

Responses of retinal arterioles were measured in vitro as previously described in detail (Gericke et al., 2018; Gericke et al., 2011). Mice were killed by CO_2_ inhalation, and the eyes were excised and placed in cold Krebs buffer. After preparation of the retina, retinal arterioles were pressurized via a micropipette placed in the ophthalmic artery by using a reservoir filled with Krebs buffer imaged with a video camera attached to a microscope (Olympus Vanox-T AH-2; Olympus Deutschland GmbH, Hamburg, Germany). The organ chamber was circulated with carbonated and oxygenated Krebs buffer at 37°C and pH 7.4. After an equilibration period of 30 min, first-order retinal arterioles were examined. First, changes in luminal diameter in response to different perfusion pressures were measured by stepwise increasing the intraluminal pressure from 10 to 80 mmHg. Subsequently, at 50 mmHg, arteriole responses to the thromboxane mimetic 9,11-dideoxy-9*α*,11*α*-methanoepoxy prostaglandin F2*α* (U46619, 10^−9^-10^−6^ M, Cayman Chemical, Ann Arbor, MI, USA) were examined. Then, responses to the endothelium-dependent vasodilator, acetylcholine (10^−8^-10^−4^ M, Sigma-Aldrich, Munich, Germany), and to the endothelium-independent NO donor, sodium nitroprusside (10^−8^-10^−4^ M, Sigma-Aldrich), were examined in vessels that had been preconstricted with U46619 to 50-70% of the initial diameter.

### 2.5. Quantification of Reactive Oxygen Species

Fluorescent dye dihydroethidium (DHE) was used to determine ROS levels *in situ* as described previously [21, 22]. After mice had been sacrificed and their eyes harvested, 10 *μ*m frozen cross sections were prepared. After thawing, the tissue sections were immediately incubated with 1 *μ*M DHE. Photographs of retinal cross sections were taken using a fluorescent microscope at an excitation wavelength of 518 nm and an emission wavelength of 605 nm. The intensity of the staining was measured in blood vessels and individual retinal layers by using ImageJ software (NIH, http://rsb.info.nih.gov/ij/) as reported previously [23, 24].

### 2.6. Quantitative PCR

Messenger RNA for the hypoxic markers, hypoxia-inducible factor 1alpha (HIF-1*α*) and vascular endothelial growth factor-A (VEGF-A), for the prooxidant redox enzymes, nicotinamide adenine dinucleotide phosphate oxidases 1 and 2 (NOX1 and NOX2), for the antioxidant redox enzymes, catalase (CAT), heme oxygenase 1 (HO-1) and glutathione peroxidases 1 and 4 (GPx1 and GPx4), for the cytokines, tumor necrosis factor alpha (TNF-*α*), interferon gamma (IFN-*γ*), interleukin 1beta (IL-1*β*), and interleukins 2 and 12 (IL-2 and IL-12), and for the nitric oxide synthase (NOS) isoforms, eNOS, iNOS, and nNOS, was quantified in the whole retinal explants as described before [24]. Tissue samples were homogenized (FastPrep; MP Biomedicals, Illkirch, France). RNA was isolated using peqGOLD TriFast™ (PEQLAB), and cDNA was generated with the High-Capacity cDNA Reverse Transcription Kit (Applied Biosystems, Darmstadt, Germany). Quantitative real-time RT-PCR (qPCR) reactions were performed on a StepOnePlus™ Real-Time PCR System (Applied Biosystems) using SYBR® Green JumpStart™ Taq ReadyMix™ (Sigma-Aldrich, Munich, Germany) and 20 ng cDNA. Relative mRNA levels of target genes were quantified using the comparative threshold (CT) normalized to the housekeeping gene TATA-binding protein (TBP). The qPCR primer sequences are shown in Table 1.

### 2.7. Immunostainings

Sagittal eye globe cryosections (10 *μ*m thickness) were incubated with rabbit polyclonal antibodies directed against NOX2 (Abcam; catalog number: ab80508, dilution 1 : 100, incubation time: 2 h at RT), HIF-1*α* (Novus Biologicals, Centennial, CO, USA; catalog number: NB 100-654; dilution 1 : 100; incubation time: 2 h at RT), or vascular endothelial growth factor-A (VEGF-A, Abcam, Waltham, MA, USA; catalog number: ab9570, dilution 1 : 100, incubation time: 2 h at RT). As a secondary antibody, a Rhodamine Red-X-coupled goat anti-rabbit polyclonal antibody (dianova GmbH, Hamburg, Germany; catalog number: 111-295-003; dilution 1 : 200, incubation time: 1 h at RT) was used. Negative control sections were incubated with blocking media and the secondary antibody only. Slides were mounted using VECTASHIELD® Mounting Medium with 4′,6-diamidino-2-phenylindole (DAPI) (BIOZOL Diagnostica Vertrieb GmbH, Eching, Germany) and cover-slipped.

### 2.8. Statistical Analysis

Data are presented as mean ± SE, and *n* represents the number of mice per group. Time courses of IOP, MAP, OPP, perfusion pressure, and concentration responses were compared using two-way repeated measures ANOVA and Sidak's multiple comparisons test. Pearson's product-moment correlation was used to test for correlation between Δ IOP and Δ OPP. For comparison of ROS levels, mRNA expression levels, and immunostaining intensity, an unpaired *t*-test was used. The significance level was set at 0.05.

## 3. Results

### 3.1. Intraocular Pressure, Blood Pressure, and Ocular Perfusion Pressure

**Figure 1:**
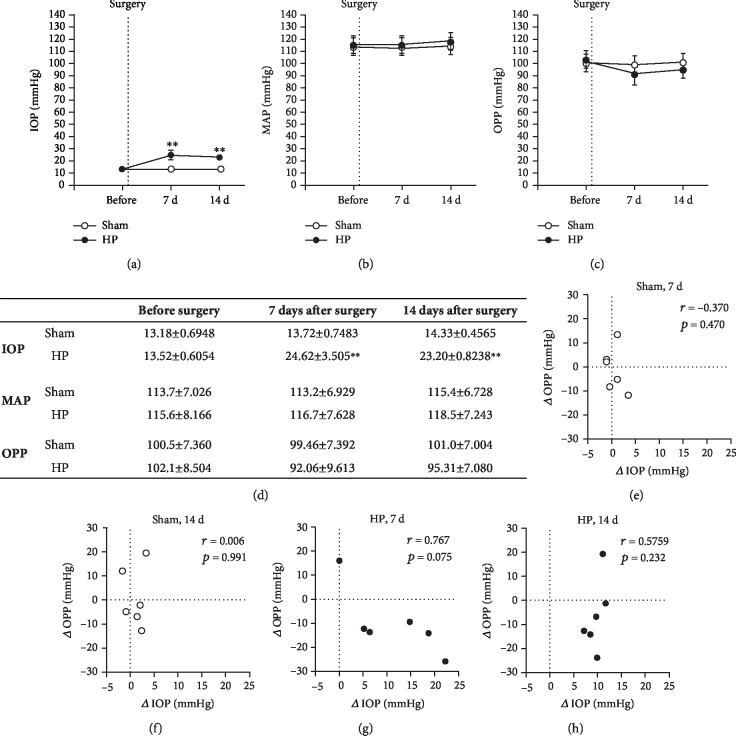
Time course of IOP (a), mean arterial pressure (b), and calculated ocular perfusion pressure (c) determined directly before as well as 7 and 14 days after surgery. The exact values are also presented (d). Scatter plot of Δ IOP and Δ OPP for the sham group 7 days (e) and 14 days (f) after surgery as well as for the high-IOP group (HP) 7 days (g) and 14 days (h) after surgery. Person's *r* and *p* values are presented in the respective figures. IOP: intraocular pressure; MAP: mean arterial pressure; OPP: ocular perfusion pressure. Values are expressed as mean ± SE (^∗∗^*p* < 0.01, *n* = 6 per group).

Before surgery, IOP was similar in both groups. However, 7 and 14 days after surgery, IOP was markedly increased in eyes that had been cauterized compared to sham-treated eyes (Figure 1). In contrast, no differences in MAP were observed between both groups. Calculated OPP did not differ between the two mouse groups either, which may be explained by the relatively small difference in IOP between cauterized and sham-treated eyes (Figures 1(a)–1(d)). Correlation analysis for Δ IOP and Δ OPP revealed only weak correlation between these two variables (Figures 1(e)–1(h)). In one mouse, OPP had increased 7 and 14 days after cauterization of episcleral veins (Figures 1(g) and 1(h), respectively), because the increase in MAP was higher than that in IOP.

### 3.2. Responses of Retinal Arterioles

**Figure 2:**
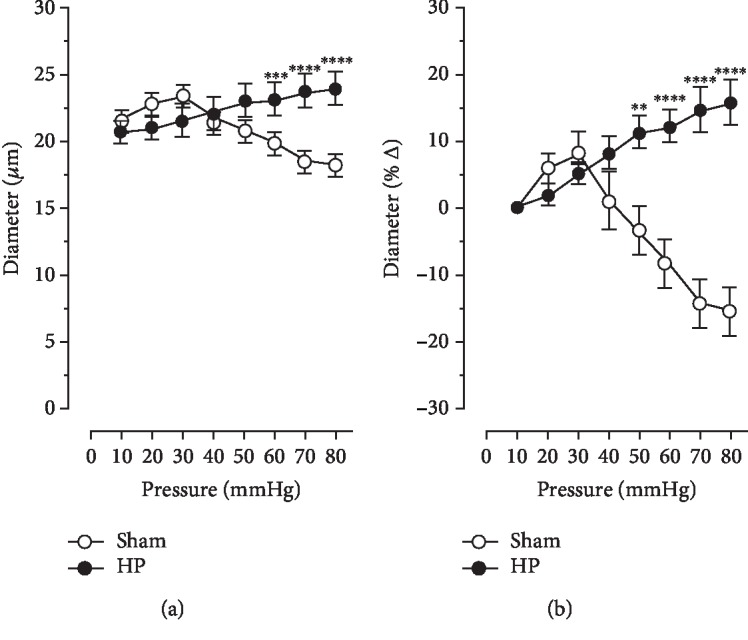
Changes of luminal diameter in retinal arterioles from high-IOP (HP) and sham-operated mice (sham) to stepwise elevation of perfusion pressure expressed as absolute diameter (a) and relative changes in diameter (b). Values are expressed as mean ± SE (^∗∗^*p* < 0.01, ^∗∗∗^*p* < 0.001, and ^∗∗∗∗^*p* < 0.0001; *n* = 6 per group).

In response to stepwise increases in perfusion pressure, retinal arterioles from sham-treated mice responded with vasoconstriction (15.83 ± 3.270% at 80 mmHg), indicative of intact autoregulation. In contrast, arterioles from mice with high IOP responded with vasodilation (15.83 ± 3.270% at 80 mmHg), suggesting that autoregulation was abnormal (Figure 2).

**Figure 3:**
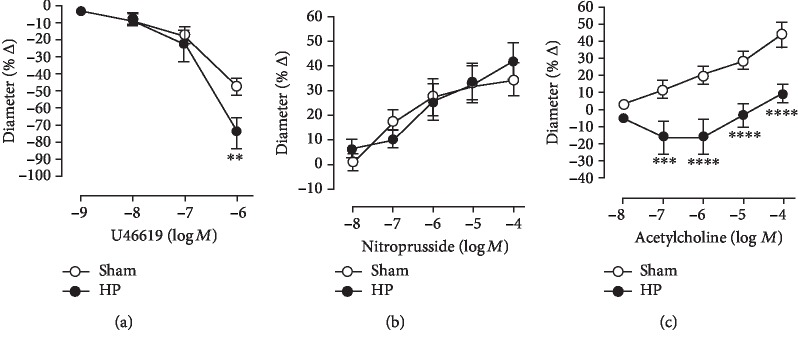
Responses of retinal arterioles to the thromboxane mimetic, U46619 (a), the endothelium-independent vasodilator, nitroprusside (b), and the endothelium-dependent vasodilator, acetylcholine (c). Values are expressed as mean ± SE (^∗∗^*p* < 0.01, ^∗∗∗^*p* < 0.001, and ^∗∗∗∗^*p* < 0.0001; *n* = 6 per group).

U46619 (10^−9^-10^−6^ M) elicited concentration-dependent vasoconstriction of retinal arterioles that was greater in mice with elevated IOP compared to sham controls. For example, reduction in luminal diameter was 46.81 ± 4.676% in sham controls and 74.50 ± 9.563% in mice with elevated IOP at 10^−6^ M (Figure 3(a)). In retinal arterioles preconstricted with U46619, the endothelium-independent vasodilator, nitroprusside (10^−8^–10^−4^ M), produced a concentration-dependent vasodilation, which did not differ between arterioles from mice with elevated IOP and sham controls. Increase in luminal diameter to 10^−4^ M nitroprusside was 34.22 ± 6.815% and 41.67 ± 7.806% in sham controls and mice with elevated IOP, respectively (Figure 3(b)). In contrast, responses of retinal arterioles to the endothelium-dependent vasodilator, acetylcholine (10^−8^–10^−4^ M), were markedly reduced in eyes with elevated IOP. For example, increase in luminal diameter to 10^−4^ M acetylcholine was 9.967 ± 4.749% and 44.10 ± 7.190% (*p* < 0.01) in sham controls and eyes with elevated IOP, respectively (Figure 3(c)).

### 3.3. ROS Levels in the Retina

**Figure 4:**
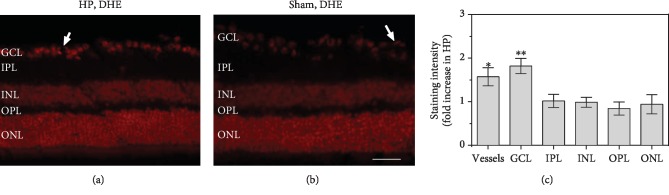
Pictures of DHE-stained retinal cryosections from mice with high IOP (HP, a) and mice that received sham surgery only (sham, b). DHE staining intensity was increased in the ganglion cell layer including blood vessels (c), but not in other retinal layers. The white arrows point to cross sections of retinal blood vessels. GCL: ganglion cell layer; IPL: inner plexiform layer; INL: inner nuclear layer; OPL: outer plexiform layer; ONL: outer nuclear layer. Values are presented as mean ± SE (^∗^*p* < 0.05, ^∗∗^*p* < 0.01; *n* = 6 per group, scale bar = 50 *μ*m).

Staining of retinal cross sections with DHE revealed markedly increased staining intensity in the retinal RGC layer and in blood vessels of high-IOP mice, indicative of increased ROS concentration (Figure 4).

### 3.4. Messenger RNA Expression of Redox and Cytokine Genes in the Retina

**Figure 5:**
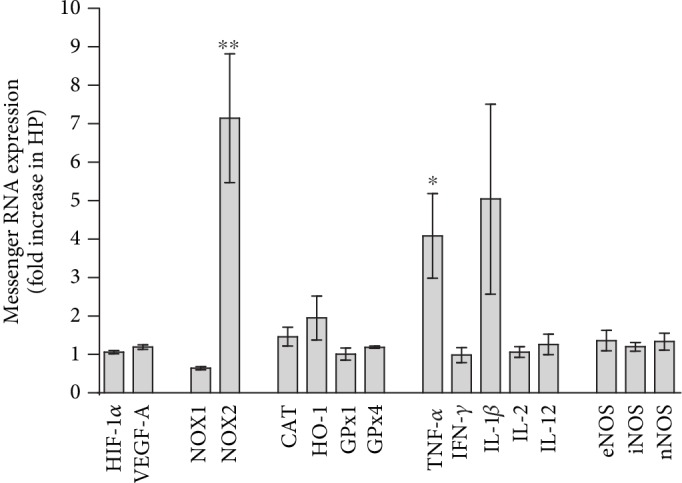
Messenger RNA expression of hypoxia genes (HIF-1*α*, VEGF-A), prooxidant genes (NOX1, NOX2), antioxidant genes (CAT, HO-1, GPx1, and GPx4), and genes coding for proinflammatory cytokines (TNF-*α*, IFN-*γ*, IL-1*β*, IL-2, and IL-12) and for nitric oxide synthases (eNOS, iNOS, and nNOS), in mice with high IOP and sham controls. Data are presented as the fold-change (mean ± SE) in eyes with high IOP (HP) versus sham controls (^∗^*p* < 0.05, ^∗∗^*p* < 0.01; *n* = 6 per group).

Messenger RNA for the hypoxia markers, HIF-1*α* and VEGF-A, and for the three nitric oxide synthase isoforms, eNOS, iNOS, and nNOS, was not increased in mice with high IOP (Figure 5). Remarkably, NOX2 expression was found to be about 7-fold higher in high-IOP mice compared to sham controls (*p* < 0.05, Figure 5). In contrast, no significant differences in mRNA expression levels of antioxidant redox genes, catalase, HO-1, GPx1, and GPx4, were found between the two groups. Among the cytokines tested, mRNA for TNF-*α* was markedly increased.

### 3.5. Expression of NOX2, HIF-1*α*, and VEGF-A in the Retina

**Figure 6:**
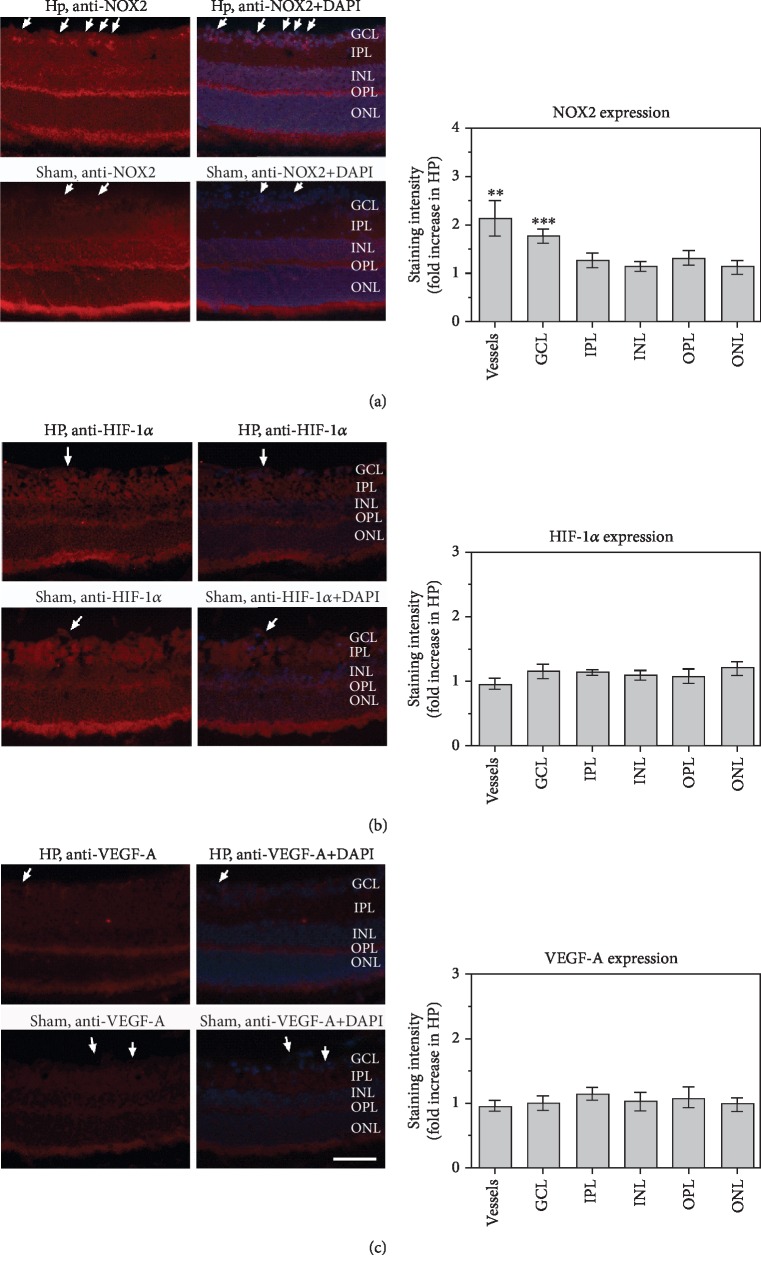
Immunostainings for NOX2 (a), HIF-1*α* (b), and VEGF-A (c) in retinal cross sections from mice with high IOP (HP) and sham controls (sham). Immunoreactivity to NOX2 was increased in retinal blood vessels and the ganglion cell layer of mice with elevated IOP but did not differ in other retinal layers of both mouse groups. Immunoreactivity to HIF-1*α* and VEGF-A was similar throughout the retina in both mouse groups. The white arrows point to retinal blood vessel cross sections. GCL: ganglion cell layer; IPL: inner plexiform layer; INL: inner nuclear layer; OPL: outer plexiform layer; ONL: outer nuclear layer. Values are presented as mean ± SE (^∗∗^*p* < 0.01, ^∗∗∗^*p* < 0.001; *n* = 6 per group, scale bar = 50 *μ*m).

While NOX2 immunoreactivity was elevated in the RGC layer and particularly in retinal blood vessels, immunoreactivity to HIF-1*α* and VEGF-A was similar in mice with normal IOP and elevated IOP (Figure 6).

## 4. Discussion

There are several new findings in the present study. First, experimental unilateral elevation of IOP had no effect on blood pressure and OPP. However, elevated IOP blunted retinal arteriole reactivity in response to the endothelium-dependent vasodilator, acetylcholine, but not to the endothelium-independent nitric oxide donor, nitroprusside, suggesting that IOP elevation induces endothelial dysfunction. Moreover, retinal arteriole responses from mice with elevated IOP to changes of perfusion pressure were compromised, suggesting that autoregulation, a crucial adaptive mechanism to keep retinal perfusion constant, is impaired. Second, ROS levels were increased in the RGC layer and in retinal blood vessels of mice with elevated IOP. In addition, mRNA expression for the prooxidant redox gene, NOX2, and for the proinflammatory cytokine, TNF-*α*, was increased in mice with elevated IOP. Furthermore, in mice with high IOP, NOX2 expression was increased in the RGC layer and in blood vessels at the protein level underpinning its involvement in ROS generation. In contrast, the hypoxic markers, HIF-1*α* and VEGF-A, were not upregulated at the mRNA and protein levels suggesting that the hypoxic component was negligible. These data suggest that NOX2 is critically involved in the generation of retinal oxidative stress leading to endothelium-dependent vessel dysfunction at increased IOP.

Elevated IOP is a major risk factor for the onset and progression of glaucoma and currently the only treatable risk factor for the disease [5]. Observations, including the progression of glaucomatous damage even when IOP is lowered to within sometimes very low target levels, however, suggest that other factors are also crucial [5]. The present study is the first to clearly show that elevated IOP impairs retinal vascular reactivity. We have chosen an established mouse model, in which we elevated IOP by cauterization of three episcleral veins. This method was proven to induce high IOP together with RGC loss in both mice and rats [20, 25–27]. We extend the previously reported observations by demonstrating that retinal arterioles develop endothelial dysfunction and abnormal autoregulation due to elevated IOP.

Previous studies that examined the role of vascular function in glaucoma pathophysiology suggested that vasospasm, systemic hypotension, angiographic vascular perfusion defects, and alterations in blood flow parameters may result in or be a result of reduced vascular perfusion in the optic nerve head and/or retina consequently leading or at least contributing to glaucoma onset and progression [4, 28–31]. Some authors even regard normal tension glaucoma as an ocular manifestation of a systemic vascular dysfunction, as reflected in the so called Flammer syndrome [32, 33]. Other research groups found retinal vascular abnormalities in normal-tension and high-tension glaucoma patients, which include narrowing of retinal blood vessels, optic disc hemorrhage, and reduction of blood vessel density [12, 34, 35]. However, so far none of them has shown that increased IOP induces vascular dysfunction itself possibly triggering further IOP-independent RGC loss by means of vessel dysfunction.

A link between elevated IOP and impaired vascular damage in the retina may be oxidative stress. In various experimental animal models, oxidative stress was induced in retinal tissue already by moderate increases in IOP [36–38]. Interestingly, particularly the inner retinal layers, such as the RGC layer, were previously shown to be exposed to oxidative stress in ocular hypertensive eyes [38]. This finding is also supported by our findings. Oxidative stress is a leading mechanism of glutamate neurotoxicity and immune responses, which were shown to promote RGC death [39–41]. In the vasculature, oxidative stress is also a trigger factor of endothelial dysfunction in various cardiovascular diseases [42, 43]. Hence, it is reasonable to assume that oxidative stress induced by IOP elevation may also affect the reactivity of blood vessels in the retina. In support of this concept, levels of ROS and of markers for vascular endothelial function were shown to be increased in glaucoma patients both in aqueous humor and in blood serum [7–10].

A possible source of ROS in eyes with elevated IOP appears to be the prooxidant redox enzyme, NOX2, which was also upregulated in the retina of eyes with high IOP in the present study and which is known to be involved in the development of vascular disease [42]. We found that reactivity to acetylcholine, an endothelium- and eNOS-dependent vasodilator in retinal arterioles [44, 45], was compromised whereas the reactivity to the nitric oxide donor, SNP, which induces vasodilation in an endothelium-independent manner was preserved, indicative of endothelial dysfunction. This finding is in line with previous studies in humans, which reported that endothelial function and bioavailability of nitric oxide are impaired in glaucoma [46]. Our findings are also in concert with previous studies reporting that NOX2 was mediating endothelial dysfunction and endothelial cell senescence in various blood vessels, including retinal arterioles [23, 47–49]. The increased reactivity to the vasoconstrictor, U46619, observed by us may also be attributed to a lack of endothelial relaxing factors that counteract vasoconstriction. In support of this concept, we previously demonstrated that retinal arterioles with a chemically destroyed endothelium responded stronger to the vasoconstrictors, U46619 and phenylephrine [50].

Under normal conditions, the retinal vasculature possesses an intrinsic ability to maintain relatively constant blood flow despite changes in perfusion pressure, in order to meet the metabolic demands of the tissue and to avoid stress to the capillaries [51, 52]. This process, known as autoregulation, is achieved by appropriate changes in arteriolar smooth muscle tone that causes vessels to constrict or dilate in response to pressure (myogenic response), shear stress on the endothelial lining of vessels, metabolite concentrations in vessels and/or tissue, local tissue partial pressure of carbon dioxide (PCO_2_) or pH levels, and neural stimuli [53–55]. In our model, retinal arterioles of animals with elevated IOP showed abnormal responsiveness to changes of perfusion pressure, suggesting that autoregulation was compromised. While in line with previous studies retinal arterioles of the control group in a certain range responded with vasoconstriction to stepwise increases in perfusion pressure [56, 57], arterioles from mice with elevated IOP responded with diameter increases, suggesting that autoregulation was abnormal. This finding was unexpected because the vascular theory of glaucoma considers RGC death and optic nerve damage a consequence of insufficient blood supply due to vascular dysregulation and consecutive tissue hypoxia. In support of this concept, many previous studies reported on reduced calibre of retinal arterioles, reduced optic nerve head and peripapillary blood flow dynamics, and increased levels of retinal hypoxia markers, such as HIF-1*α* and VEGF, in eyes of glaucoma patients [34, 58–62]. Consequently, we would expect reduced arteriole diameters and more pronounced vasoconstriction responses due to increasing perfusion pressure as well as an increase in retinal hypoxic markers, such as HIF-1*α* and VEGF-A, in eyes with elevated IOP. However, the fact that we found increasing vessel diameters and no changes of hypoxic markers speaks against the hypothesis of reduced perfusion and hypoxia at least at the initial stage of glaucoma development.

Based on our findings, we hypothesize that the autoregulative response is switched off or at least reduced in the retinal vasculature as an adaptive mechanism to protect retinal tissue from ischemia or lack of nutrients due to increased vasoconstriction, which would be expected when endothelium-dependent vasodilation is impaired. Since autoregulation is a local adaptive response of blood vessels to a certain level of intravascular pressure, it is very likely that our measurements obtained in the isolated retina reflect also the autoregulative responses in vivo. As a consequence of impaired autoregulation, the retina may become hyperperfused resulting in an increase in capillary pressure, which consecutively may lead to damage of capillaries with consequent tissue ischemia/hypoxia. In support of this hypothesis, recent studies utilizing optical coherence tomography (OCT) angiography reported that capillary density and blood flow were reduced in the retina and optic nerve of glaucoma patients [13–15]. Moreover, previous histopathological studies in human retinal tissue revealed that expression of the O_2_ sensing molecule, HIF-1*α*, is increased in glaucoma and is concordant with the location of visual field defects [58]. Based on our findings, it is unlikely that hypoxia was a trigger factor for the observed impaired vascular reactivity because expression of the hypoxic markers, HIF-1*α* and VEGF-A, was not elevated when IOP was increased. However, it may be possible that hypoxia may become a result of secondary vascular changes at later stages of the disease. Since both mouse groups had similar MAP, their different retinal arteriole reactivity cannot be attributed to differences in blood pressure. Elevated arterial blood pressure is a well-known trigger factor of endothelial dysfunction in various blood vessels, including ocular and cerebral vessels [63, 64]. Moreover, elevated arterial pressure and low OPP have been associated with an increased risk of glaucoma [65]. The fact that we did not achieve a significant reduction of OPP by IOP elevation may be explained by the only moderate IOP difference between both mouse groups.

Regarding the choice of our glaucoma model, we do not expect direct effects of episcleral vein cauterization on retinal arteriole responses. Although cauterization may induce heat in the peripheral retina as seen by the clumping of the retinal pigment epithelium at the site of heat exposure, vascular responses were measured more centrally, thus making it unlikely that these vascular segments were exposed to heat. Moreover, direct heat exposure to a vessel would very likely induce a functional deterioration of the vascular smooth muscle as well, which was not the case, because responses to U46619 and to SNP were not impaired in cauterized eyes.

## 5. Conclusions

The findings of our study are particularly striking because they reveal for the first time that elevated IOP causes endothelial dysfunction in ocular blood vessels. Of note, the IOP increase was only moderate in the present study and did not cause marked changes in OPP. Hence, the intriguing question arises regarding the mechanism. One possibility is that oxidative stress is induced by elevated IOP in neurons or glial cells, as previously shown, and transmitted to adjacent vascular cells via more stable ROS forms, such as hydrogen peroxide, or indirectly via proinflammatory cytokines, such as TNF-*α*, and inflammatory cells. However, it is also possible that ROS are directly induced in vascular cells by elevated IOP. Since HIF-1*α* and VEGF-A expression was not increased in the retina including retinal blood vessels, endothelial dysfunction and impaired autoregulation do not appear to induce ischemia in retinal tissue. However, the findings do not exclude the possibility that hypoxia occurs at a later stage of glaucoma. One intriguing question that remains to be solved is whether endothelial dysfunction and abnormal autoregulation will persist once the IOP gets back to normal levels, since this might provide an answer to the question as to why glaucoma is still progressing in patients even when the IOP is therapeutically held within normal limits.

## Figures and Tables

**Table 1:** Primer sequences used for quantitative PCR analysis.

Gene	Forward	Reverse
HIF-1*α*	TCATCAGTTGCCACTTCCCCAC	CCGTCATCTGTTAGCACCATCAC
VEGF-A	ACTTGTGTTGGGAGGAGGATGTC	AATGGGTTTGTCGTGTTTCTGG
NOX1	GGAGGAATTAGGCAAAATGGATT	GCTGCATGACCAGCAATGTT
NOX2	CCAACTGGGATAACGAGTTCA	GAGAGTTTCAGCCAAGGCTTC
CAT	CAAGTACAACGCTGAGAAGCCTAAG	CCCTTCGCAGCCATGTG
HO-1	GGTGATGCTGACAGAGGAACAC	TAGCAGGCCTCTGACGAAGTG
GPx1	CCCGTGCGCAGGTACAG	GGGACAGCAGGGTTTCTATGTC
GPx4	GCA TCC CGC GAT GAT TG	TCG ATG TCC TTG GCT GAG AAT
TNF-*α*	GCC TCT TCT CAT TCC TGC TTG	CTG ATG AGA GGG AGG CCA TT
IFN-*γ*	AGC GGC TGA CTG AAC TCA GAT TGT AG	GTC ACA GTT TTC AGC TGT ATA GGG
IL-1*β*	AAG GAG AAC CAA GCA ACG ACA AAA	TGG GGA ACT CTG CAG ACT CAA ACT
IL-2	CAA GTC CTG CAG GCA TGT ACA	CTG TTG ACA AGG AGC ACA AGT GT
IL-12	CAT CCA GCA GCT CCT CTC AGT	GCA AGG GTG GCC AAA AAG A
eNOS	CCTTCCGCTACCAGCCAGA	CAGAGATCTTCACTGCATTGGCTA
iNOS	CAGCTGGGCTGTACAAACCTT	CATTGGAAGTGAAGCGTTTCG
nNOS	TCCACCTGCCTCGAAACC	TTGTCGCTGTTGCCAAAAAC
TBP	CTT CGT GCA AGA AAT GCT GAA T	CAG TTG TCC GTG GCT CTC TTA TT

## Data Availability

The datasets used to support the findings of this study are available from the corresponding author upon reasonable request.

## Abbreviations

CAT: Catalase
CT: Comparative threshold
DAPI: 4′,6-Diamidino-2-phenylindole
DHE: Dihydroethidium
GPx: Glutathione peroxidase
HIF-1*α*: Hypoxia-inducible factior-1*α*
HO-1: Heme oxygenase
IFN-*γ*: Interferon gamma
IL: Interleukin
IOP: Intraocular pressure
MAP: Mean arterial pressure
NOS: Nitric oxide synthase
NOX: Nicotinamide adenine dinucleotide phosphate oxidase
OPP: Ocular perfusion pressure
RGC: Retinal ganglion cells
ROS: Reactive oxygen species
TBP: TATA-binding protein
TNF-*α*: Tumor necrosis factor alpha
U46619: 9,11-Dideoxy-9*α*,11*α*-methanoepoxy prostaglandin F2*α*
VEGF-A: Vascular endothelial growth factor-A.
